# Determinants of severe dehydration from diarrheal disease at hospital presentation: Evidence from 22 years of admissions in Bangladesh

**DOI:** 10.1371/journal.pntd.0005512

**Published:** 2017-04-27

**Authors:** Jason R. Andrews, Daniel T. Leung, Shahnawaz Ahmed, Mohammed Abdul Malek, Dilruba Ahmed, Yasmin Ara Begum, Firdausi Qadri, Tahmeed Ahmed, Abu Syed Golam Faruque, Eric J. Nelson

**Affiliations:** 1 Department of Medicine, Stanford University School of Medicine, Stanford, CA, United States of America; 2 Division of Infectious Diseases, Department of Medicine, University of Utah School of Medicine, Salt Lake City, UT, United States of America; 3 Division of Microbiology and Immunology, Department of Pathology, University of Utah School of Medicine, Salt Lake City, UT, United States of America; 4 International Centre for Diarrhoeal Disease Research, Bangladesh (icddr,b), Dhaka, Bangladesh; 5 Emerging Pathogens Institute and Child Health Research Institute, Department of Pediatrics, College of Medicine, University of Florida, Gainesville, FL, United States of America; 6 Department of Environmental and Global Health, College of Public Health and Health Professions, University of Florida, Gainesville, FL, United States of America; Christian Medical College, INDIA

## Abstract

**Background:**

To take advantage of emerging opportunities to reduce morbidity and mortality from diarrheal disease, we need to better understand the determinants of life-threatening severe dehydration (SD) in resource-poor settings.

**Methodology/findings:**

We analyzed records of patients admitted with acute diarrheal disease over twenty-two years at the International Centre for Diarrhoeal Disease Research, Bangladesh (1993–2014). Patients presenting with and without SD were compared by multivariable logistic regression models, which included socio-demographic factors and pathogens isolated. Generalized additive models evaluated non-linearities between age or household income and SD. Among 55,956 admitted patients, 13,457 (24%) presented with SD. *Vibrio cholerae* was the most common pathogen isolated (12,405 patients; 22%), and had the strongest association with SD (AOR 4.77; 95% CI: 4.41–5.51); detection of multiple pathogens did not exacerbate SD risk. The highest proportion of severely dehydrated patients presented in a narrow window only 4–12 hours after symptom onset. Risk of presenting with SD increased sharply from zero to ten years of age and remained high throughout adolescence and adulthood. Adult women had a 38% increased odds (AOR 1.38; 95% CI: 1.30–1.46) of SD compared to adult men. The probability of SD increased sharply at low incomes. These findings were consistent across pathogens.

**Conclusions/significance:**

There remain underappreciated populations vulnerable to life-threatening diarrheal disease that include adult women and the very poor. In addition to efforts that address diarrheal disease in young children, there is a need to develop interventions for these other high-risk populations that are accessible within 4 hours of symptom onset.

## Author summary

To best help patients with diarrheal disease, we need to better understand what causes patients to tip from mild disease to severe disease. In this study, we analyzed records of patients presenting to one of the largest hospitals in the world dedicated to the management of diarrheal diseases. Patients with and without severe dehydration (SD) were compared at multiple-levels. We found that: (i) Patients presented with SD after only 4 to 12 hours of symptoms. (ii) The risk of presenting with SD was higher in older children and adult women; suggesting that people might seek help differently based on age and gender. (iii) The extreme poor had a dramatically increased risk of presenting with SD. In summary, these findings highlight the need for rapid interventions that target older children and adult women in addition to ongoing efforts focused on young children.

## Introduction

Medical professionals face difficult decisions on how best to triage patients in high-volume, resource-limited settings. This is especially true during large-scale diarrheal disease outbreaks that can quickly overwhelm first-responders and manifest in high mortality rates [1]. Understanding the determinants of life-threatening diarrheal disease will enable better identification of vulnerable populations that can be targeted early at both the level of the hospital and community.

Our understanding of vulnerable populations to diarrheal disease has been shaped by a historical emphasis of combating diarrheal disease among children less than 5 years of age. While diarrheal disease is responsible for one in ten deaths among children less than 5 years of age and necessitates ongoing attention [2–5], there are important knowledge gaps in our understanding of the burden of severe diarrheal disease in older populations [6–8]. Large studies have yielded critical new insights into the microbial determinants of diarrheal disease and its severity among children [9–11]; however, there are limited data on the microbial determinates of severe disease among adolescents and adults in developing countries [6, 12, 13].

In this study, we asked how we might best leverage one of the largest databases on diarrheal disease in the world, housed at the International Centre for Diarrhoeal Disease Research, Bangladesh (icddr,b) [14, 15], to begin to fill in these gaps at the level of the hospital. We expanded on previous studies that looked at in-hospital mortality by comparing patients presenting at hospital-admission with and without severe dehydration [16]. Using data covering a twenty-two year period and non-parametric methodologies, we examined the influence of age, sex, and socioeconomic status in the context of high-priority pathogens on the risk of severe dehydration due to diarrheal disease.

## Methods

### Ethics statement

The DDSS has been approved by the Research Review Committee (RRC) and Ethical Review Committee (ERC) at the icddr,b; the Stanford University institutional review board determined that it did not require additional approval. The DDSS has been described previously in detail [15, 17]. In brief, at admission, verbal consent is taken from adult patients, or from adult caregivers or guardians on behalf of children or adult patients that are unable to provide consent. Consenting individuals are assured non-disclosure of personal health information and informed that data is used to improve patient care activities and facilitate research that may be conveyed by publication; subjects may refuse participation without compromise to patient care. The ERC has approved this verbal consent procedure and is satisfied with the voluntary participation, maintenance of the rights of the participants and confidential handling of personal information by the hospital physicians.

### Study design and participants

This study was conducted at the main icddr,b hospital in the capital city of Dhaka, Bangladesh. Dhaka has 15 million inhabitants and is burdened with high rates of diarrheal disease. At the icddr,b, the Diarrheal Disease Surveillance System (DDSS) prospectively collects demographic, economic, clinical, and enteric pathogen data from every 25^th^ (1993–1995) or 50^th^ (1995-present) diarrheal patient [14, 15]. The study design was a retrospective case-control study of DDSS patients from 1993 to 2014. Inclusion criteria were patients of all ages admitted with acute diarrheal disease (three or more loose stools per 24 hours). ‘Acute’ was defined as an onset within the previous 7 days, which aligns with previous studies [9, 10].

### Outcomes

Cases were participants presenting with severe dehydration (SD) and controls were those presenting without SD (‘no’ or ‘some’ dehydration). Dehydration status was classified by the admitting doctor, or triage nurse, at hospital admission. The clinical classifications of ‘no’, ‘some’, and ‘severe’ dehydration approximate 0–4%, 5–9%, and 10% weight loss, respectively. These classifications are made using the icddr,b ‘Dhaka Method’ [18] that is derived from WHO guidelines [19]. The Dhaka Method differs in that it includes radial pulse and the scoring method is more robust yet more cumbersome. In the Dhaka Method, ‘Some’ dehydration requires at least two findings of restlessness/irritability, sunken eyes, drinks eagerly/ thirsty, and skin pinch goes back slowly (2 to less than 3 second); at least one must be restlessness/irritability and/or drinks eagerly/ thirsty. SD occurs when the criteria for ‘some’ dehydration’ are met plus at least one key finding in the highest category of lethargy/unconscious, skin goes back very slowly (> = 3 sec), drinks poorly/ unable to drink, or uncountable/ absent pulse. In the WHO guidelines, dehydration status is scored according to having two clinical signs in the highest category. A standardized questionnaire was administered to each patient to ascertain demographic and socioeconomic information, as well as clinical history; treatment received (rehydration method, antibiotics) and clinical outcomes (mortality, discharge status) were reported in a standardized database.

### Laboratory procedures

A stool sample from each patient underwent standard culture-based isolation techniques for enteric pathogens [20]. In brief, *V*. *cholerae* were isolated by growth on tellurite taurocholate gelatin agar (TTGA) media with enrichment in bile peptone broth followed by antisera panel testing (Denka Seiken Co., Ltd.) for Ogawa or Inaba antigens and 2.5% chicken cell agglutination tests were performed for phenotypic characterization (e.g. for El Tor and Classical), *Salmonella* spp. and *Shigella* spp. were isolated by growth on MacConkey agar and Salmonella-Shigella (SS) agar with enrichment in selenite broth followed by antisera panel testing (Denka Seiken Co., Ltd.), *Campylobacter* spp. were isolated by growth on Brucella *agar*, and *Aeromonas* spp. were isolated by growth on TTGA and gelatin agar followed by phenotypic characterization of long-sugar metabolism. Enterotoxigenic *Escherichia coli* (ETEC) was detected by standard methods [21, 22]; these assays were performed from 1996 to 2001 by enzyme-linked immunosorbent assays (ELISA) and 2007 to 2014 by both ELISA and polymerase chain reaction (PCR). Rotavirus was detected by ELISA [23]. Assays for *Campylobacter* spp. and *Aeromonas* spp. were not performed between 2002–2008. Assays for additional pathogens (e.g. cryptosporidium, norovirus) were not routinely performed.

### Statistical analysis

Demographic, economic, and clinical characteristics were described by proportions for categorical variables and median with interquartile ranges (IQR) for continuous data. Because income increased during the 22 year study period, and relative poverty was of key interest, we created a Z score for income that was normalized by year (subtracting the annual mean and dividing by the standard deviation). We also constructed a household asset Z score using principal components analysis (PCA) and normalized the score by year; PCA components included number of rooms in the household, number of beds, access to electricity, access to gas for cooking, and ownership of a fan, television, low-quality cot, and high-quality cot. Although the diagnostic assays identified many pathogens, the analysis focused on 7 pathogens (*Vibrio cholerae*, Rotavirus, ETEC, *Aeromonas* spp., *Shigella* spp., *Campylobacter* spp., and non-typhoidal *Salmonella*) because they were both the most common pathogens identified and had been previously identified as high-priority pathogens associated with symptomatic disease at hospital admission [9, 10].

For the primary analysis comparing participants with and without SD, we used bivariable and multivariable logistic regression models, with SD as the dependent variable, and expressed the results as odds ratios (OR) and adjusted odds ratios (AOR) for SD. Because of the large number of observations, we retained all variables in the initial multivariable analysis. We examined pathogen interaction by fitting multivariable logistic regression models, adjusted for age and sex, with each combination of the seven pathogens; the odds of SD in mono-infection were compared with the odds in the presence of the second pathogen using a generalized linear hypothesis test. To evaluate non-linear risk relationships with key predictors of SD, we utilized generalized additive models with logistic link functions with penalized regression splines [24, 25]. Generalized additive models were compared with multivariable logistic regression models using Akaike Information Criteria, which balances model fit and parameter number [26], and Chi-square testing. We additionally performed multivariable linear regression to evaluate independent predictors of time to presentation from the onset of symptoms. All analyses were performed using R (R Foundation for Statistical Computing)[27].

## Results

**Fig 1:**
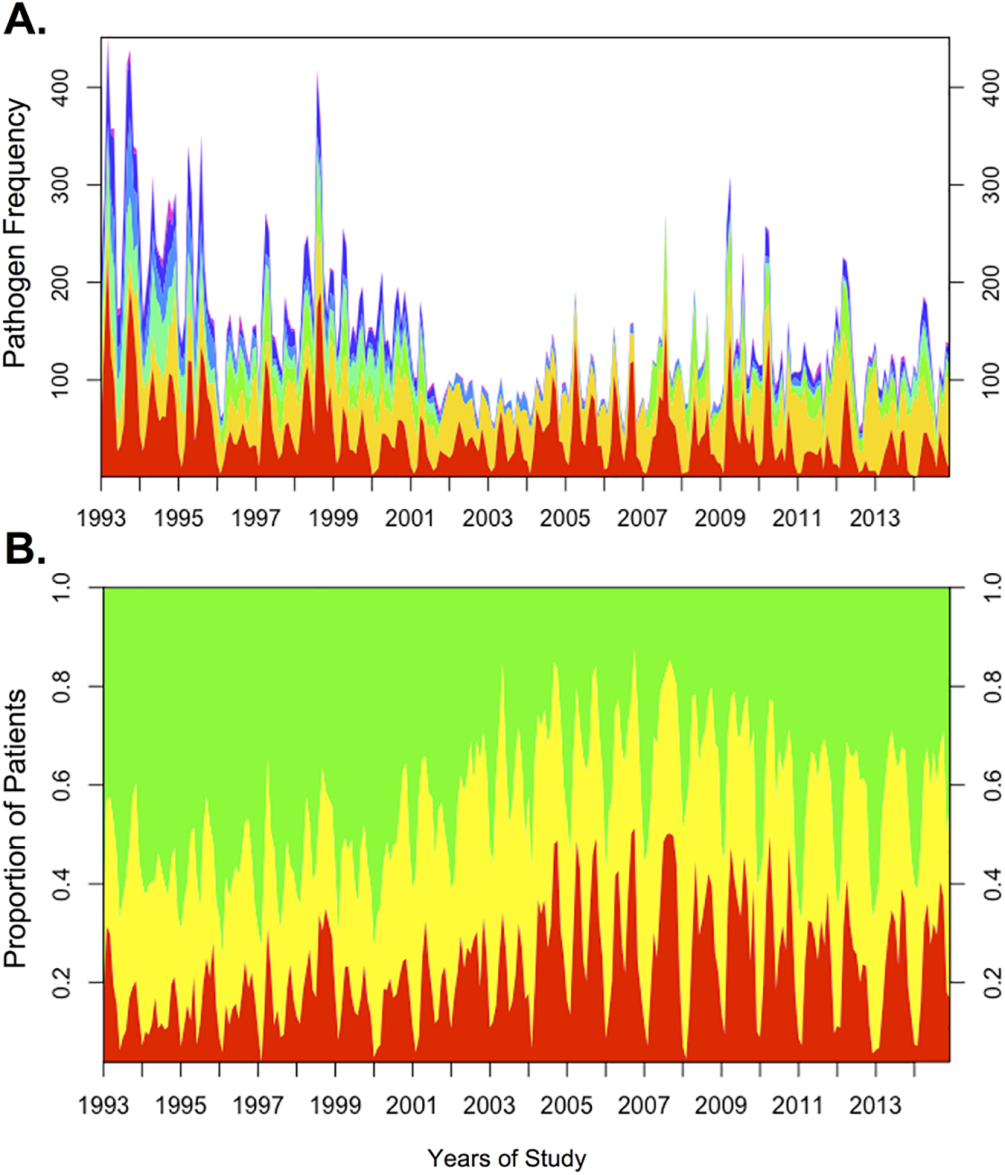
**(A)** Monthly frequency of pathogens isolated from 1993–2014. Red = *V*. *cholerae;* yellow = rotavirus; green = ETEC; blue = *Aeromonas* spp.; *Shigella* spp. = purple; *Campylobacter* spp. = orange. Non-typhoidal *Salmonella* = black. Surveillance enrollment rates were 4% for 1993–1995 and 2% for 1996–2014. **(B)** Proportion of patients presenting with severe (red), some (yellow), and no (green) dehydration across the study period.

**Fig 2:**
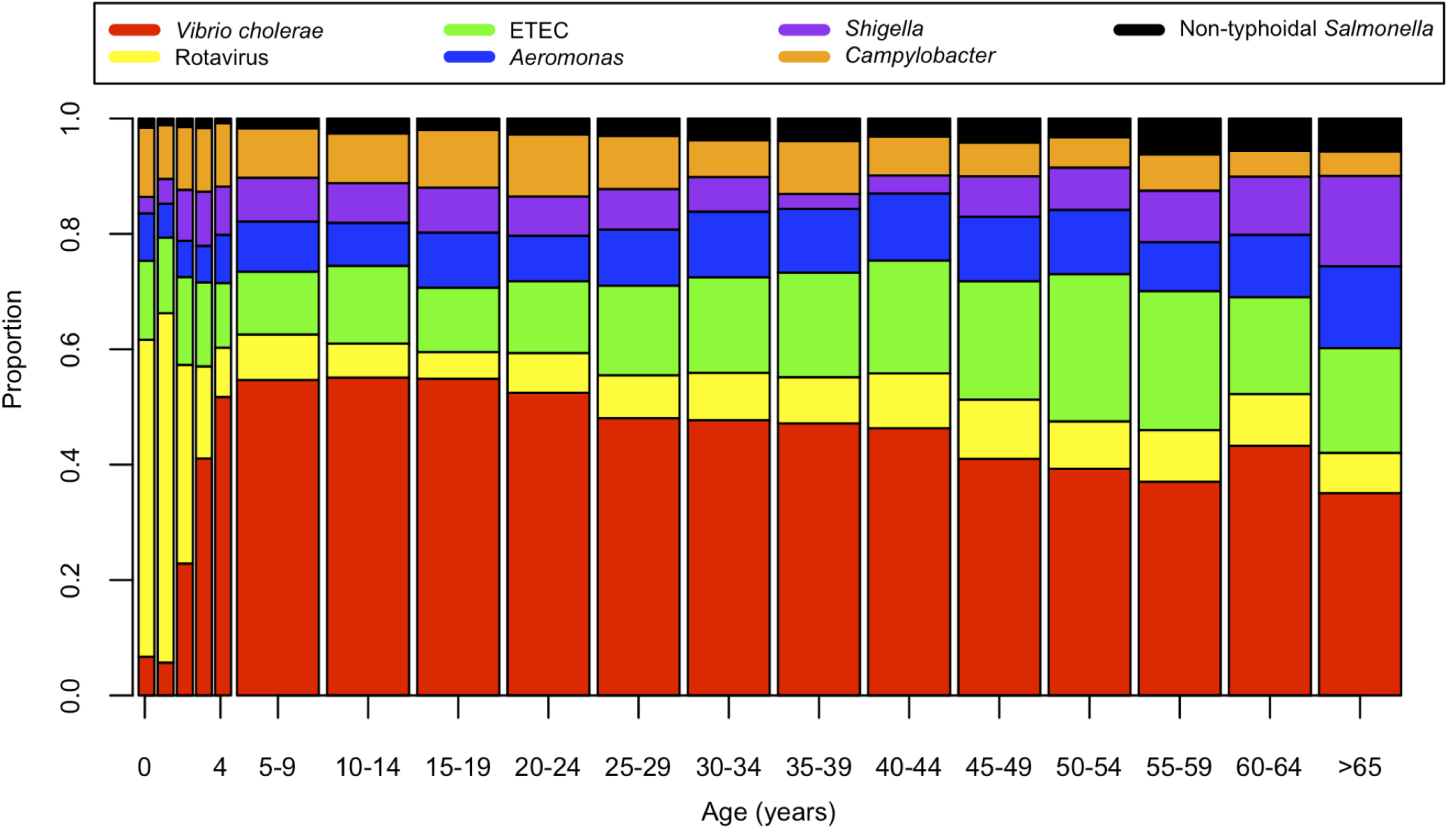
Distribution of high-priority pathogens identified as a function of patient age.

During the 22-year surveillance period, 55,956 patients met criteria for analysis (Table 1). Among all participants, 41% were female and 52% were under 5 years of age. The most common pathogens identified were: *V*. *cholerae* (12,405; 22%), rotavirus (11,959; 21%), ETEC (3,582 of 32,741 tested; 11%), *Aeromonas* spp. (3,248 of 41,550 tested; 8%), *Shigella* spp. (3,162; 6%), *Campylobacter* spp. (3,365 of 41,550 tested; 8%) and Non-typhoidal *Salmonella* (825; 1%; Table 1). In total, 62% of patients had at least one of these pathogens identified from a stool sample. Consistent with previous studies, the distribution of these pathogens varied over the 12-month period (Fig 1A and S1 Fig) with an increase of *V*. *cholerae* and decrease of rotavirus between the Spring and Fall periods for each study year. The biannual distribution of cholera outbreaks (Figs 1A and S1) correlated with a rise in the proportion of patients presenting with severe dehydration for each study year. When controlling for multiple factors including *V*. *cholerae* isolation, the proportion of patients presenting with severe dehydration was highest in later years (2004–2014) (Table 1). When aggregated, the distribution of pathogens varied by age (Fig 2). *V*. *cholerae* was the most common pathogen identified in all age groups, except children ages 0–2 years, in whom rotavirus was most common. Twelve percent of patients had more than one pathogen detected in their stools. The number of pathogens identified per patient was independent of household income.

**Table 1:** Population characteristics and microbiologic findings for individuals presenting with and without severe dehydration.

			Dehydration Status						
		All Patients	None/Mild	Severe					
		N = 55,956	N = 42499 (76%)	N = 13457 (24%)	OR	p	AOR	CI	p
Population characteristics									
Age, years; N (%)									
	0–4	28866 (52)	27210 (64)	1656 (12)	ref	-	ref	ref	-
	5–9	2879 (5)	1790 (4)	1089 (8)	10.0	<0.0001	6.2	(5.31–7.24)	<0.0001
	10–14	1908 (3)	1035 (2)	873 (6)	13.68	<0.0001	9.12	(7.70–10.80)	<0.0001
	15–19	2844 (5)	1465 (3)	1379 (10)	14.47	<0.0001	11.58	(10.02–13.38)	<0.0001
	≥20	19459 (35)	10999 (26)	8460 (63)	12.64	<0.0001	11.85	(10.81–13.01)	<0.0001
Sex, Female; N (%)		22997 (41)	16706 (39)	6291 (47)	1.36	<0.0001	1.18	(1.10–1.26)	<0.0001
Socio-economic status (SES)									
	Family Income Z-score; mean (SD)	0 (1)	0.07 (1.06)	-0.21(0.73)	0.64	<0.0001	0.70	(0.66–0.73)	<0.0001
	Household Asset Score	0 (1)	0.10	-0.30	0.67	<0.0001			
	Children in household; median (IQR)	1 (0–1)	1 (1–1)	1 (0–1)	0.41	<0.0001			
Spatial, temporal, and longitudinal									
	Distance in miles; median (IQR)	7 (5–13)	6.5 (5–13)	7 (5–12)	1.00	<0.0001	1.01	(1.00–1.01)	<0.0001
	Time traveled, minutes; median (IQR)	50 (30–90)	45 (30–90)	50 (30–90)	1.00	0.0136			
	Night onset (7pm-7am); N (%)	20949 (45)	15225 (44)	5724 (48)	1.15	<0.0001	1.08	(1.01–1.16)	0.026
	1993–1997; N (%)	17364 (31)	14559 (84)	2805 (16)	ref	<0.0001			
	1998–2003; N (%)	12271 (22)	9650 (79)	2621 (21)	1.41	<0.0001			
	2004–2008; N (%)	10880 (19)	7207 (66)	3673 (34)	2.65	<0.0001			
	2009–2014; N (%)	15441 (28)	11083 (72)	4358 (28)	2.04	<0.0001			
Pathogens detected; N (%)									
	*Vibrio cholerae*	12405 (22)	5257 (12)	7148 (53)	8.03	<0.0001	4.77	(4.41–5.15)	<0.0001
	Rotavirus	11959 (21)	11504 (28)	455 (3)	0.09	<0.0001	0.42	(0.37–0.48)	<0.0001
	Enterotoxigenic *E*. *coli* (ETEC)*	3582/32741 (11)	2719/24371 (11)	863/8370 (10)	0.92	0.032	1.08	(0.97–1.20)	0.171
	*Aeromonas* spp.**	3248/41550 (8)	2714/32590 (8)	534/8960 (6)	0.70	<0.0001	0.77	(0.67–0.90)	<0.001
	*Shigella* spp.	3162 (6)	2853 (7)	309 (2)	0.33	<0.0001	0.43	(0.35–0.52)	<0.0001
	*Campylobacter* spp.**	3365/41550 (8)	2750/32590 (8)	615/8960 (7)	0.80	<0.0001	0.91	(0.79–1.05)	0.196
	Non-typhoidal *Salmonella*	825 (1)	682 (2)	143 (1)	0.66	<0.0001	0.65	(0.50–0.84)	0.001
Number of pathogens detected per patient; N (%)§									
	0	10609 (38)	8182 (39)	2427 (36)	ref	—			
	1	13670 (49)	10176 (49)	3494 (52)	1.16	<0.001			
	2	2997 (11)	2272 (11)	725 (11)	1.08	0.132			
	≥ 3	362 (1)	299 (1)	63 (1)	0.71	0.015			

SD-Standard deviation. IQR-Interquartile range. OR-Odds ratio. AOR-Adjusted odds ratios. CI-Confidence interval. ref-Reference.

The multivariable model excluded household asset score, the time traveled prior to admission, and pathogen number.

* Gaps in years of data collection (1993–1995 and 2002–2006).

** Gaps in years of data collection (2002–2008).

§ Calculated for years 1996–2001 and 2009–2014, when all pathogens were tested (N = 27638).

At hospital presentation, the most common symptoms were watery stools (94%) and vomiting (79%) (Table 2). The median duration of diarrhea prior to admission was 27 hours (IQR: 12–56 hours). Patients with SD presented earlier after the onset of symptoms (13 hours vs 34 hours; p<0.001), reported higher stool counts, and more often reported watery diarrhea (99% vs 92%, p<0.001) and vomiting (95% vs 74%, p<0.001). Reported ORS use prior to presentation was very high (90–91%) in both groups. Severely dehydrated patients more often trialed IV solution (11% vs 4%; p<0.001) and were less likely to report antibiotic use (22% vs 41%; p<0.001); both are readily available at community pharmacies.

**Table 2:** Hospital presentation and course in individuals presenting with and without severe dehydration.

				Dehydration Status			
			All Patients	None/Mild	Severe		
			N = 55,956	N = 42499	N = 13457	OR	p
Hospital Presentation							
Hours of diarrhoea prior to admission; median (IQR)			27 (12–56)	34 (16–67)	13 (8–24)	0.97	<0.0001
Number of loose stools in 24 hours; N (%)							
	3–5		4881 (9)	4072 (10)	809 (6)	ref	<0.0001
	6–10		23634 (42)	19538 (46)	4096 (30)	1.06	0.202
	11–15		15615 (28)	11081 (26)	4534 (34)	2.06	<0.0001
	16–20		6461 (12)	4324 (10)	2137 (16)	2.49	<0.0001
	>20		5364 (10)	3483 (8)	1881 (14)	2.72	<0.0001
History of; N (%)							
	Watery stool		52432 (94)	39047 (92)	13385 (99)	16.43	<0.0001
	Bloody stool		1687 (3)	1639 (4)	48 (0.4)	0.09	<0.0001
	Vomiting		44445 (79)	31660 (74)	12785 (95)	6.51	<0.0001
	Use of oral rehydration solution (ORS)		50603 (90)	38320 (90)	12283 (91)	1.14	<0.0001
	Use of IV fluid		3027 (5)	1522 (4)	1505 (11)	3.39	<0.0001
	Use of antibiotics		12793/34566 (37)	11067/26696 (41)	1726/7870 (22)	0.40	<0.0001
Measurements (6 months to 5 years); N (%)							
	Mid-upper Arm Circumference (<115mm)		2366/22400 (12)	2204/21150 (10)	162/1250 (13)	1.28	0.005
Hospital course							
	Initial rehydration with IV fluids; N (%)		16440 (29)	3474 (8)	12966 (97)	318.95	<0.0001
	Duration of admission in hours; median (IQR)		14 (5–27)	12 (4–27)	18 (9–28)	1.00	<0.0001
	Mortality; n (%)		148 (0.26)	107 (0.25)	41 (0.30)	1.21	0.340
		Age <5	113/28828 (0.39)	90/27175 (0.33)	23/1653 (1.39)	4.25	<0.0001
		Age ≥5	35/27073 (0.13)	17/15278 (0.11)	18/11795 (0.15)	1.37	0.350

IQR-Interquartile range. OR-Odds ratio. ref-Reference. See methods for definition of dehydration status.

A total of 13,457 (24%) patients presented with SD and 42,499 patients presented without SD. Presentation with SD was associated with older age (median age 25 vs 1.5 years in patients without SD; AOR 1.03 per year, 95% CI: 1.03–1.04) and female sex (AOR 1.18, 95% CI: 1.10–1.26). Among children, severe malnutrition, assessed by mid-upper arm circumference [28], was associated with a modest increased odds (OR 1.28) of presenting with SD (Table 2). *V*. *cholerae* strongly associated with presentation with SD (AOR: 4.77, 95% CI: 4.41–5.15), ETEC and *Aeromonas* spp. had a balanced distribution between patients with and without SD and *Shigella* spp., *Campylobacter* spp., rotavirus and non-typhoidal *Salmonella* spp. were negatively associated with SD (Table 1). We further evaluated the association between specific pathogen combinations and disease severity in multivariate analysis (Table 3). Among the 21 dual-pathogen combinations, none were associated with a higher risk of SD compared with the more severe (higher OR) of the two single-pathogen infections. Three combinations (all involving *Aeromonas* spp.) were associated with a lower risk of SD than either single-pathogen infection. Although this study focuses on events prior to admission, mortality rates were low and not significantly different between patients with and without SD (0.30% vs 0.25%; p = 0.34). Consistent with previous studies [7], mortality was highest among children less than 5 years of age (Table 2; S2 Fig). The duration of admission was longer for patients with SD (18 vs 12 hours; p<0.001).

**Table 3:** Age- and sex-adjusted probability of presenting with severe dehydration as a function of single or dual-pathogen combinations.

Patients with one or at least two pathogens detected in stool (N*)								
	*Vibrio cholerae*	Rotavirus	ETEC		*Aeromonas*	*Shigella*	*Campylobacter*	Non-typhoidal *Salmonella*
*Vibrio cholerae*	10174	479	536		199	267	800	109
Rotavirus		9530	691		531	283	666	109
ETEC			1920		253	111	312	32
*Aeromonas* spp.					1825	275	339	56
*Shigella* spp.						2133	219	31
*Campylobacter jejuni*							1313	38
Non-typhoidal Salmonella								508
**Probability of presenting with severe dehydration in the setting of one or two pathogens detected**								
** **	*Vibrio cholerae*	Rotavirus		ETEC	*Aeromonas*	*Shigella*	*Campylobacter*	Non-typhoidal *Salmonella*
*Vibrio cholerae*	**0.72**	0.69		0.76	0.60	0.63	0.69	0.60
Rotavirus		**0.26**		0.45	0.24	0.28	0.17	0.38
ETEC				**0.56**	0.43	0.49	0.47	0.48
*Aeromonas* spp.					**0.30**	0.11**	0.19**	0.13**
*Shigella* spp.						**0.21**	0.25	0.31
*Campylobacter jejuni*							**0.44**	0.27
Non-typhoidal Salmonella								**0.32**

* For the dual-pathogen combinations, the stool samples contained at least the stated two pathogens; rare samples had more than 2 pathogens detected.

** Statistically significant (p < 0.05) for a decreased probability of SD than either single-pathogen infection (bold text).

No dual-pathogen combinations were associated with a significant increase in SD compared to the more severe of the two single-pathogen infections.

**Fig 3:**
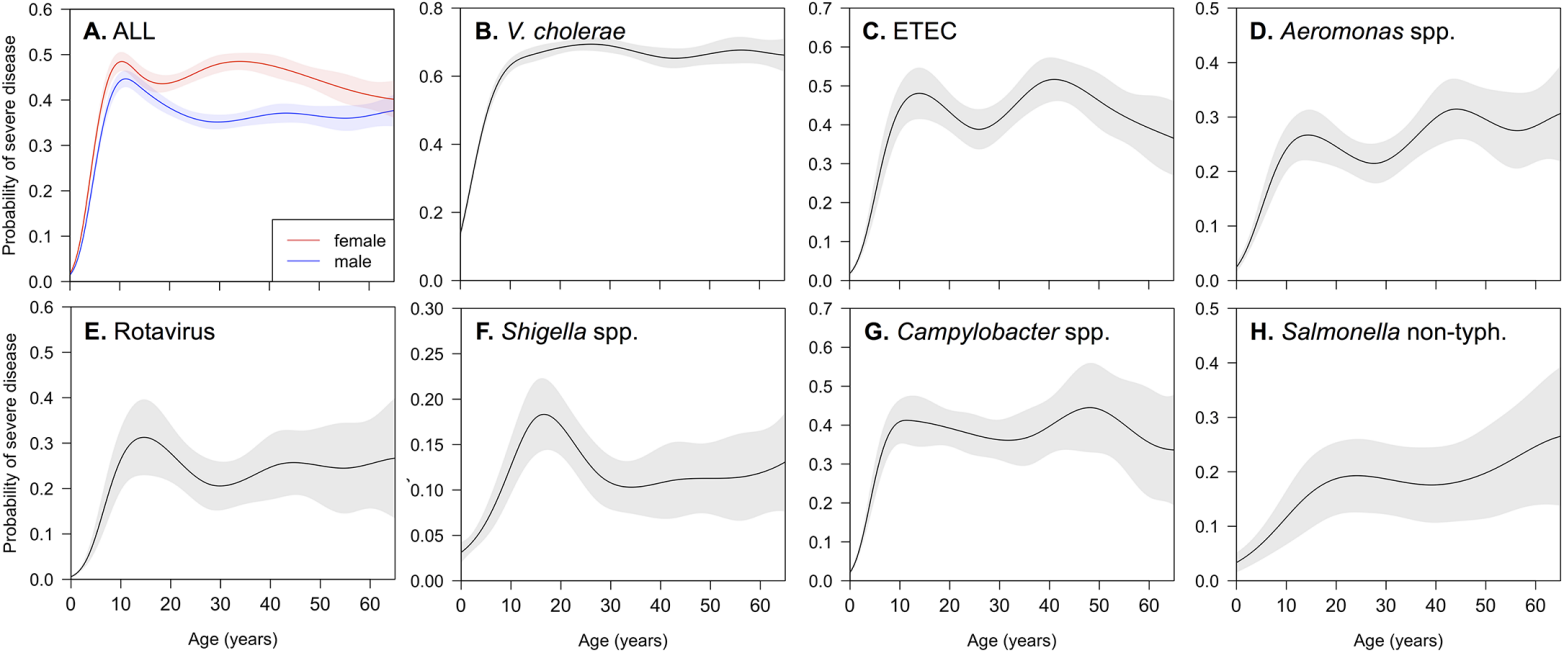
Predicted probability of severe dehydration as a function of age, for all patients (A) or patients with specific pathogens (B-H). Panel A shows sex-stratified age-specific risks for all patients (male = blue, female = red). Shading represents the 95% confidence interval.

**Fig 4:**
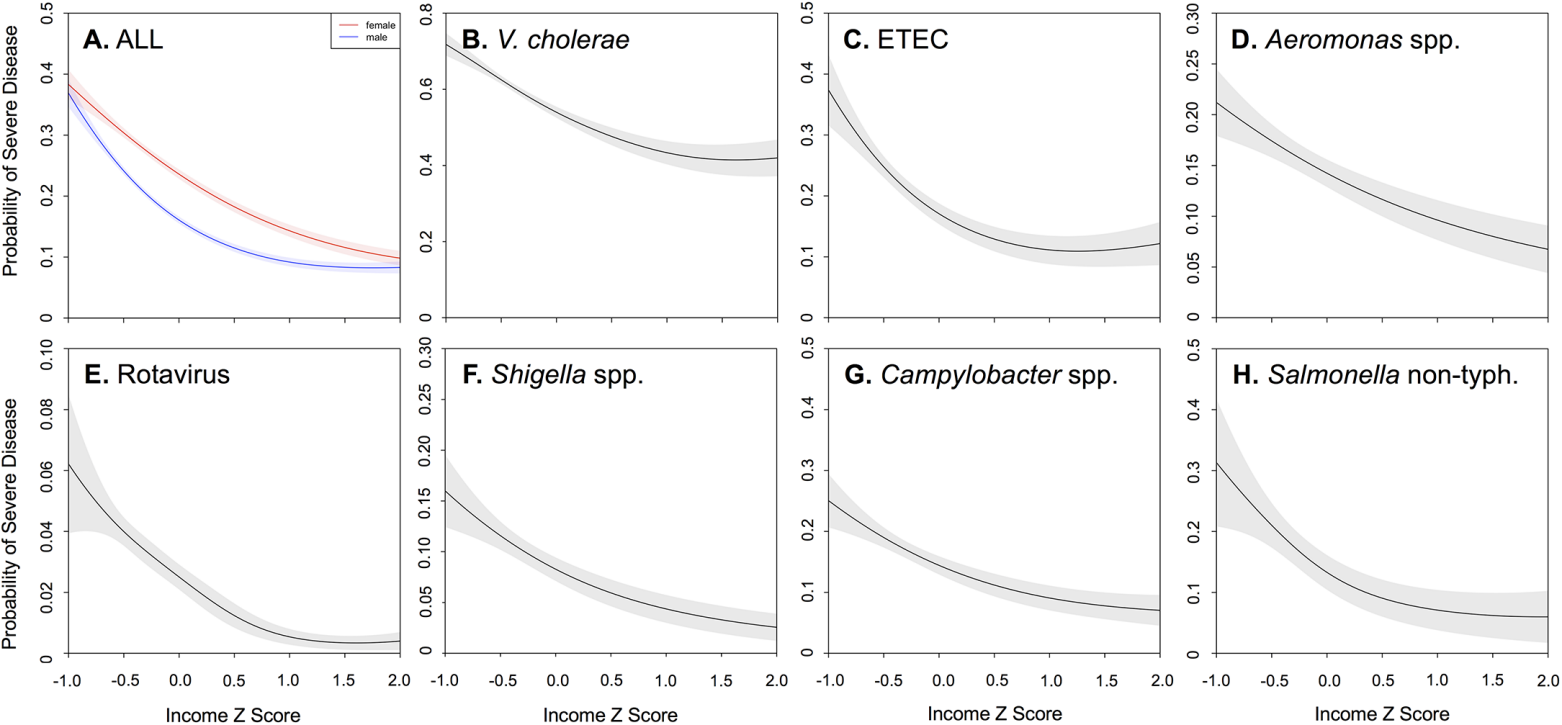
Predicted probability of severe dehydration as a function of family income Z score, by pathogen. Panel A shows sex-stratified risks as a function of income Z score for all patients (male = blue, female = red). Shading represents the 95% confidence interval. Remaining panels (B-H) show risk as a function of income Z score for specific pathogens.

Multivariable logistic regression identified age, gender, and income as critical determinants of SD. We then used generalized additive models to assess potential non-linearities in the relationship between these key variables and risk of SD. We found that the relationship between age and risk of SD was strongly non-linear; the risk rose steeply between ages 0 and 10 (Fig 3). While the probability of SD was similar for the sexes among children, it bifurcated between 15 and 20 years of age, with adult women experiencing a 38% increased odds of SD (AOR 1.38; 95% CI: 1.30–1.46) compared with adult men. Although the relative scale varied, the findings were generally consistent across pathogens. The probability of presenting with SD was also determined as a function of household income (Fig 4) and household asset score (Table 1). For all pathogens, the probability of SD increased sharply at low incomes (Z scores 0 to -1), with adjusted odds ratios 2–10 times that at the highest quintile. These non-linear effects were most dramatic for ETEC and rotavirus (Fig 4).

**Fig 5:**
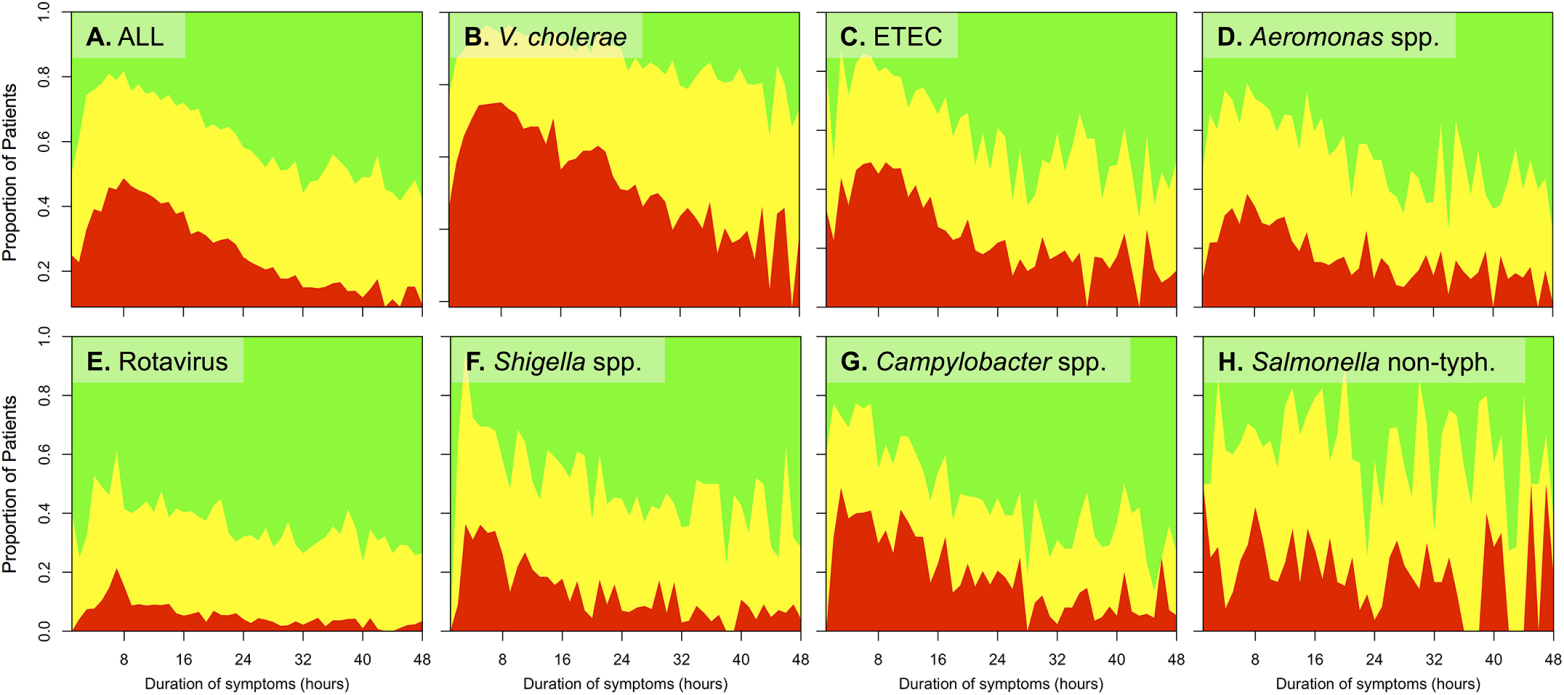
Dehydration status as a function of symptom duration prior to presentation. The proportion of patients with severe (red), some (yellow), and no (green) dehydration are shown for all patients (A) or patients with a specific pathogen (B-H).

The highest proportion of patients with SD presented in a narrow window four to twelve hours after symptom onset, with a peak at approximately 8 hours (Fig 5). With the exception of non-typhoidal *Salmonella*, the highest proportion of patients with SD was observed soon after symptom onset, and this fraction declined rapidly over subsequent hours. In multivariable analyses, SD was inversely associated with a longer duration of symptoms prior to presentation (β = -12.2 hours, SE = 0.4, p<0.001), while age less than 5 years was positively associated with a longer duration of symptoms prior to presentation (β = 21.5 hours, SE = 0.3, p<0.001; S3 Fig). Female sex and income were not associated with the duration of symptoms prior to admission (β = 0.1 hours, SE = 0.3, p = 0.823; β = 0.0 hours, SE = 0.1, p = 0.764, respectively).

## Discussion

In a database of over 55,000 patients presenting with acute diarrheal disease to the icddr,b over a period of 22 years who underwent systematic clinical and microbiologic investigations, we identified several important insights into the determinants of presenting with life-threatening dehydration from diarrheal disease. First, we found that the risk of presenting with SD was much higher in older children and adolescents than in younger children (<5 years), and that adult women had a 38% increased odds of presenting with SD compared with adult men. Second, the risk of SD was inversely proportionate to income, and this relation was non-linear, with risk increasing dramatically among the extreme poor. Importantly, these demographic findings held true across the most common seven pathogens. Finally, while multiple pathogens were identified in 20% of participants in whom a pathogen was identified, we found no increased risk of SD in these cases. In addition to existing efforts focused on children less than 5 years of age, these findings highlight the need to develop additional rapid interventions targeting older children and adult women to further prevent severe morbidity and mortality from diarrheal disease.

The identification of vulnerable sub-populations not previously appreciated provides additional insight that the clinical evaluation, pathophysiology and epidemiology of diarrheal disease is more complicated than previously thought. This new era of inquiry was sparked by two landmark studies (GEMS and MAL-ED) that ranked pathogens associated with symptomatic diarrheal disease among young children, helped identify a sub-set of high-value pathogens for vaccine development and control, and revealed that healthy young children often harbor a significant burden of pathogens yet are asymptomatic [9, 10]. Despite these advances, there remained key questions about how multi-pathogen infection, age, gender, and SES put patients at risk of severe disease that the DDSS could help answer. We first hypothesized that patients with two pathogens compared to one pathogen would have an increased probability of presenting with SD. For the 21 pathogen-pairs tested, we found no combination associated with an increased probability of SD. Three interactions, all with *Aeromonas* spp., were statistically associated with decreased probability of SD. It is possible that disease severity or antimicrobials influenced co-pathogen detection, and the inclusion of broader quantitative approaches (e.g. qPCR) might identify pathogens present but not assayed (e.g. cryptosporidium, norovirus). Nevertheless the results raise important questions about pathogen interaction and pathophysiology.

The sequence of events that precipitate hospital admission are complex. We found that individuals with SD presented soon after symptom onset. There was a strikingly narrow window between four to twelve hours after symptom onset that correlated with the highest probability of SD. After this window, symptom duration correlated with a decreased probability of SD; these findings were similar across pathogens. The interdependence between severe diarrhea driving both rapid help-seeking and development of SD makes mechanistic inquiry difficult. Disentangling these factors at a single time-point (e.g. hospital admission) is unlikely and requires combined community and hospital studies.

Although just over half of patients presenting at the hospital were less than five years of age, older children, adolescents and adults had the highest probability of presenting with SD, with a dramatic increase per year of life between 1 and 10 years. Why older patients were more likely to present with SD and earlier, yet have lower mortality, is an intriguing open question. One possibility is that there are survival biases among young children and adults who die of severe disease prior to presentation, such that those who reach care are more likely to have less severe disease compared with older children and adult women. However, perhaps a more important behavioral explanation, consistent with previous findings [8], is that the threshold for adults to seek care was too high and mild adult cases, particularly among women, were not represented at the hospital level. If true, this is an opportunity for high-impact community-based education and targeted intervention. A measure of outreach success might be a decrease in the proportion of older patients presenting with SD. One physiologic explanation may be found in the differences in how and when adults and children manifest signs of hypovolemic shock [29]. Adults have less physiologic reserve and vascular compliance that results in earlier appreciation of signs of uncompensated shock (e.g. poor perfusion resulting in altered mental status). In contrast, children have more physiologic reserve and vascular compliance that results in uncompensated shock manifesting later. These differences could drive differential helping seeking behavior for adults and children, and explain higher mortality rates in children seen previously [8]. Future hospital and community studies that include broader pediatric and adult specific endpoints (e.g. behavioral and physiologic) are needed to answer these questions. Finally, these studies may also reveal that using the same method to assess dehydration for adults and children is not appropriate, and an alternative scoring method that accommodates for differences between children and adults is needed [30, 31].

We found that sex did not significantly impact the risk of presenting with SD in young patients. However, adult women were 38% more likely to present with SD compared to men. The explanation may not simply be delay in seeking care because duration of symptoms prior to admission was independent of sex. In addition, household income alone cannot explain the finding because almost all women, except for the extremes, were at increased risk compared to men. We hypothesize that gender-based sociologic inequalities likely impact this risk more than gender-based differences in pathogen exposure or pathophysiologic response. Future studies will need to delineate these hypotheses.

Although not a new finding, poverty strongly correlated with SD across pathogens. However, the statistical approach taken revealed a particularly strong correlation between SD and extreme poverty. Because the distribution was right skewed, when normalized, very few people had Z scores less than -1; the majority of the population fell within 0 to -1 standard deviations of the mean, and it was in this range that the poverty relationship with SD was very strong and steep; analysis using a household asset index yielded consistent results. Whether this relationship, which was pathogen-independent, was a function of host biological factors (immune compromise; malnutrition; environmental enteropathy), care-seeking behaviors, or other factors merits further investigation. Our findings indicate that even relatively small but targeted interventions among the extremely poor may substantially reduce the incidence of SD.

Case fatality rates remain low at iccdr,b, and only 148 deaths were reported among over 55,000 patients reported in this surveillance database. This limited the power with which to investigate specific pathogen and host determinants of mortality risk. As noted above, we found higher case fatality rates among young children despite lower rates of severe dehydration. In multivariable analysis, mortality rates were lowest in rotavirus (AOR 0.14, p<0.0001) and cholera (AOR 0.33, p = 0.002) and highest in *Salmonella* (AOR 1.59, p = 0.43) and *Shigella* (AOR 1.60, p = 0.10) infections. No differences were seen between the sexes.

These findings should be viewed within the context of the limitations of the study design and available data. First, these data are from patients presenting to a single, large center; while a diverse array of infections, ages, and socioeconomic classes were represented, the associations age, sex and income with SD should be investigated in other settings to evaluate the generalizability and external validity of these findings. Second, the definition of SD relies on a clinical assessment algorithm. Classification bias is possible with algorithms despite their common use [32, 33]. In particular, whether classification differs between age groups is of importance in understanding whether the age effects could be explained by differential classification. A portion of previous studies use IV fluid solution administration as a proxy for disease severity [33, 34]. We chose to compare patients with and without SD because vomiting may necessitate IV fluids in patients without SD; that said, we were reassured that IV fluid use correlated well with clinical assessment; 1%, 18%, and 97% of patients with no, some, and severe dehydration received IV fluids, respectively. We found both positive, negative and null associations between specific pathogens and risk of severe dehydration; we do not intend to imply that these pathogens cannot cause severe dehydration, but rather that relative to cholera (the most common pathogen), most pathogens are associated with lower risk. This was a hospital-based study, and selection bias remains a critical consideration, because patients of different demographics and disease severity may seek hospital care unequally. It is possible that certain demographic groups only sought care for severe disease, and for mild disease, sought care in the community or elsewhere (or not at all) [8]. Similarly, individuals who died prior to presentation to the hospital were not captured in this analysis, and their demographic and microbiologic characteristics may differ from those that reached care. The study design focused on seven pathogens that were both the most common pathogens isolated and identified as actionable high-priority pathogens based on recent literature [9, 10]. Other pathogens could not be reliably detected with the methods used in this surveillance system, however may still be relevant and important determinants of severe dehydration. Newer molecular diagnostics may yield additional insights concerning pathogen determinants of severe dehydration. Finally, this was a case-control analysis of a large observational dataset, and subject to potential biases inherent to that design; however, we would note that the data collection instrument was prospectively administered to patients at the time of their diarrheal episode. Data collection was thereby performed consistently and in the same manner throughout the 22 year surveillance period, reducing risks of classification bias or differential data ascertainment.

Despite these limitations, the results from this study can be applied to efforts to better risk-stratify admitted patients, now with considerations for adolescence and adults of female gender. In addition, campaigns to development improved dehydration assessment algorithms should also consider these groups. At the community-level, these findings will help guide the design of much needed studies to characterize patients that do not seek hospital admission. These studies may lead to educational interventions addressing ‘why’ and ‘when’ to seek care for diarrheal disease, especially for adolescents and adult women.

In conclusion, this study provides new insights into the determinants of life-threatening SD from diarrheal disease, identifying older children, adolescents and adult women as high-risk groups for SD from diarrheal disease; additionally, we found that poverty was strongly associated with risk of presenting with SD, and that this effect was accelerated at the lowest incomes. Importantly, these findings held true irrespective of the causative pathogen. Further research is needed to understand the mechanisms behind these risk factors, whether they are host factors or treatment-seeking differences, and to refine existing and deploy emerging interventions to aid these vulnerable populations.

## Supporting information

S1 Fig Monthly frequency of high-priority pathogens identified in patient stools across the 22-year study period. Red = *V. cholerae;* yellow = rotavirus; green = ETEC; blue = *Aeromonas* spp.; *Shigella* spp. = purple; *Campylobacter* spp. = orange. Non-typhoidal *Salmonella* = black. Surveillance enrollment rates were 4% for 1993–1995 and 2% for 1996–2014.
(TIFF)

S2 Fig Case fatality rate during hospital admission as a function of age. Shading represents the 95% confidence interval.
(TIFF)

S3 Fig Cumulative proportion of individuals admitted to the hospital as a function of symptom duration. Stratification is by age **(A)** and dehydration severity **(B)**.
(TIFF)
