# Supplementary figures and images for: Determinants of severe dehydration from diarrheal disease at hospital presentation: Evidence from 22 years of admissions in Bangladesh
Source: PLoS Negl Trop Dis. 2017 Apr 27;11(4):e0005512. doi: 10.1371/journal.pntd.0005512 (PMC5423662; doi:10.1371/journal.pntd.0005512)

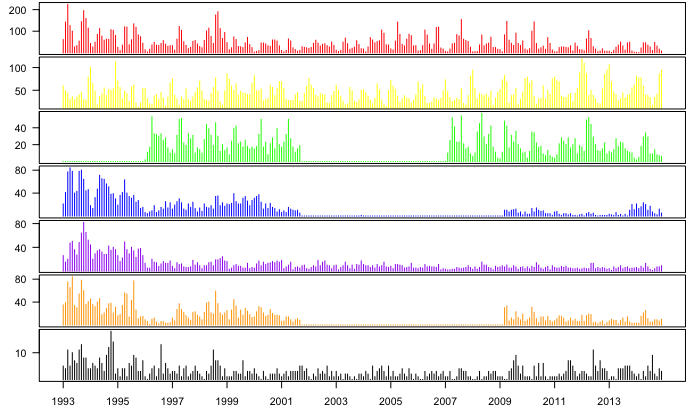

Supplement: S1 Fig — Red = V. cholerae; yellow = rotavirus; green = ETEC; blue = Aeromonas spp.; Shigella spp. = purple; Campylobacter spp. = orange. Non-typhoidal Salmonella = black. Surveillance enrollment rates were 4% for 1993–1995 and 2% for 1996–2014. (TIFF) [file pntd.0005512.s001.tiff]

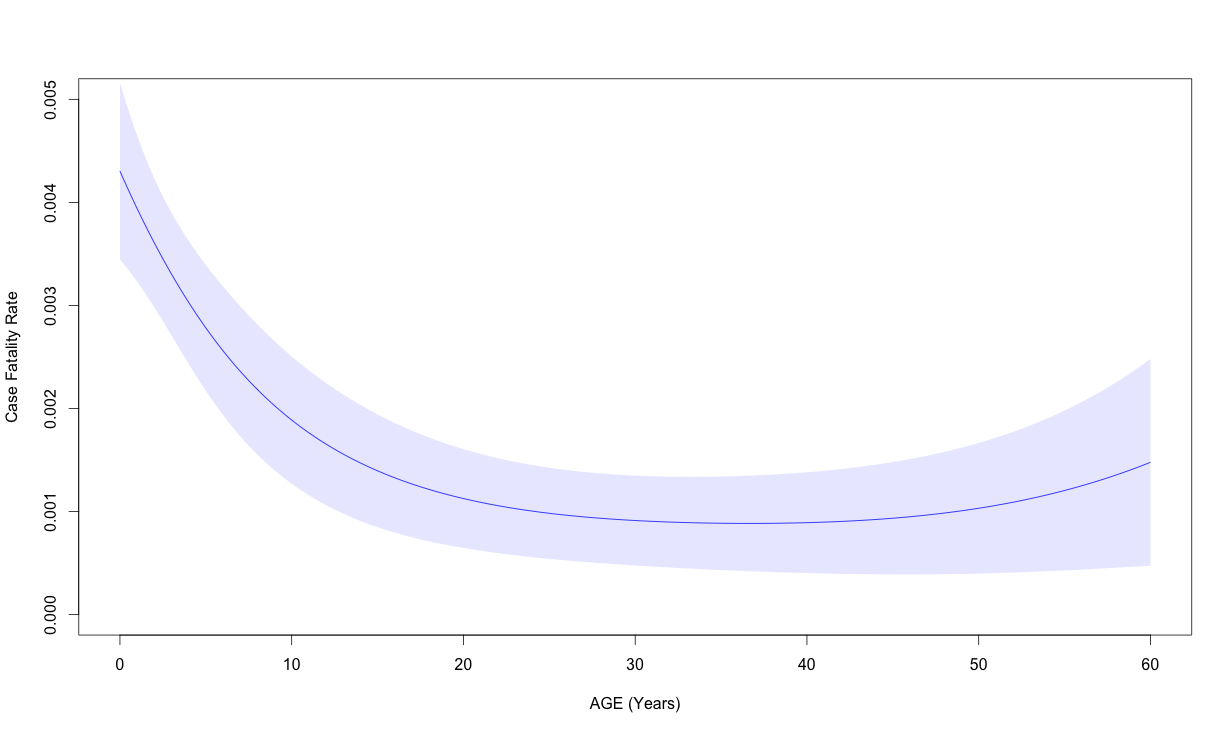

Supplement: S2 Fig — Shading represents the 95% confidence interval. (TIFF) [file pntd.0005512.s002.tiff]

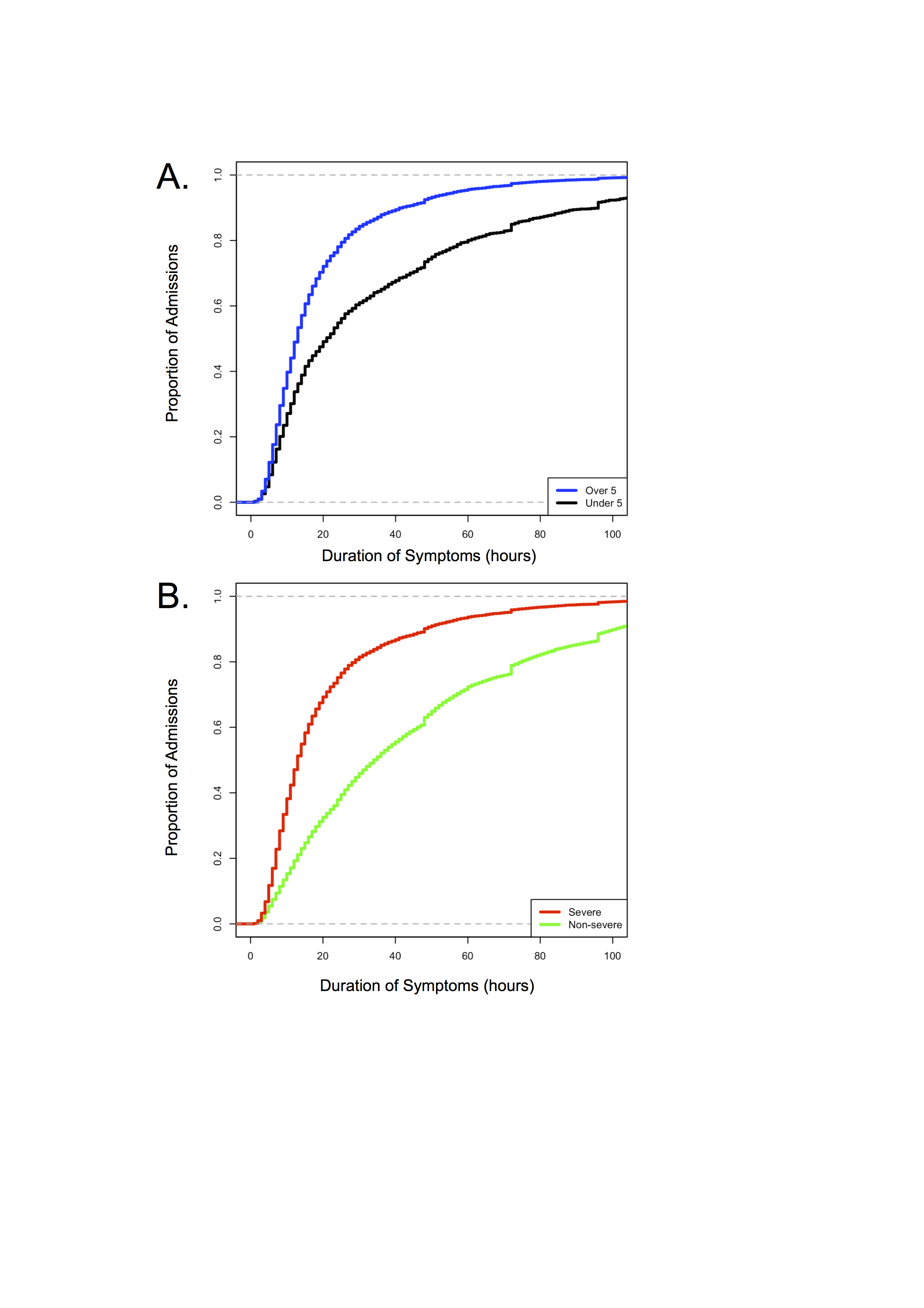

Supplement: S3 Fig — Stratification is by age (A) and dehydration severity (B). (TIFF) [file pntd.0005512.s003.tiff]
